# Personal

**Published:** 1915-03

**Authors:** 


### PERSONAL.

---

Announcement of removal, travel, and other matters of interest are requested. Please report errors in the listing of any physician in the State and other directories, that we may co-operate with the State Society in securing a correct list.

---

Dr. Arthur Hilton Paine has opened an office for genito-urinary practice at 268 Alexander Street, Rochester.

---

Dr. James A. MacLeod, of Buffalo, spent February at Pinehurst.

---

Dr. Wm. Warren Britt, of Tonawanda, who was reported seriously injured in an automobile accident last month, has recovered entirely.

Dr. J. C. Clemesha, of Buffalo, left for a southern trip on February 14.

Dr. J. Sperans, of Buffalo, has moved from 949 Clinton Street to 268 Fillmore Avenue.

Dr. Alice Schley, of Buffalo, has been appointed a manager of the Gowanda State Hospital of which she was formerly a resident physician.

Dr. W. E. Herbert, of Wellington, N. Z., describes in the New Zealand Medical Journal, a trip around the world. He pays well deserved compliments to Dr. George W. Crile, of Cleveland, and the Mayor of Rochester, Minn.

Dr. DeWitt G. Wilcox, of Boston, formerly of Buffalo, visited Buffalo early in February.

Dr. Charles J. Mengis, of Buffalo, is in California, expecting to return about April 1.

Dr. James S. Porter, of Buffalo, has moved to 36 Hedley Place

